# Accounting for the placebo effect of surgery in surgical trials?

**DOI:** 10.1186/1745-6215-14-S1-P11

**Published:** 2013-11-29

**Authors:** David Beard, Cushla Cooper, Ines Rombach, Jon Rees, Karolina Wartolowska, Naomi Cummings, Andrew Carr

**Affiliations:** 1 University of Oxford, Oxford, UK

## 

Compared to pharmacology, the efficacy of surgery has been very poorly evaluated. Many surgical procedures are carried out on a daily basis without any good evidence to support them. Primarily this is because the methodology for surgical trials offers some unique challenges. Whilst a comparison between two (or more) surgical procedures is possible, such a model only provides information on relative efficacy. It does not address the fundamental issue of whether the treatment provides any more benefit than the significant placebo effect obtained from undergoing a surgical procedure. A placebo controlled comparison is therefore preferred, but there are several ethical, implementation and interpretation obstacles to overcome before the design can be accepted as standard.

Using a current example of a placebo controlled surgical trial of shoulder surgery (CSAW), a number of issues surrounding placebo controlled surgical trials will be explored. These will include; which designs can be used to account for the placebo effect, what constitutes "placebo" surgery, how to identify and manipulate the "critical surgical element" (CSE) and operational problems associated with placebo surgical trials.

The objective of the paper is to share recent experiences and derived knowledge to help with future placebo controlled surgical trial design.

